# Potential Cryptic Diversity in the Genus *Scoliodon* (Carcharhiniformes: Carcharhinidae): Insights from Mitochondrial Genome Sequencing

**DOI:** 10.3390/ijms252111851

**Published:** 2024-11-04

**Authors:** Peiyuan Ye, Yuanxiang Miao, Chen Wang, Pichai Sonchaeng, Sarawut Siriwong, Shaobo Chen, Junjie Wang, Xiao Chen

**Affiliations:** 1 College of Marine Sciences, South China Agriculture University, Guangzhou 510642, China; cuiluoshi1998@163.com; 2 Sino-Thai Research Center of Marine Technology, Zhejiang Mariculture Research Institute, Wenzhou 325000, China; pichai.s@samudhabhipat.org (P.S.); csrbrsmeng@163.com (S.C.); 3 School of Life Science and Technology, ShanghaiTech University, Shanghai 201210, China; miaoyx1@shanghaitech.edu.cn; 4 College of Ocean and Earth Sciences, Xiamen University, Xiamen 361012, China; wangchen2971@163.com; 5 Faculty of Marine Technology, Burapha University, Thamai, Chantaburi 22170, Thailand; sarawuts@go.buu.ac.th; 6 Guangzhou Key Laboratory of Subtropical Biodiversity and Biomonitoring, School of Life Science, South China Normal University, Guangzhou 510631, China

**Keywords:** *Scoliodon*, mitochondrial genome, cryptic species, machine learning

## Abstract

*Scoliodon* is a genus of small placental sharks living in offshore waters. For a long time, the genus was considered a monotypic genus until a valid species, *Scoliodon macrorhynchos*, was confirmed in 2010. However, *S. muelleri* in the same study was not widely recognized because of the lack of evidence. In this study, we obtained the complete mitochondrial genome of the genus *Scoliodon* from Ranong, Thailand, and tentatively named it *Scoliodon* sp. By comparing the complete mitochondrial genome with those of two other *Scoliodon* species and conducting phylogenetic and divergence time analyses, we determined that *Scoliodon* sp. diverged from the other species. These findings indicate the potential for a new cryptic species (*Scoliodon* sp.) in the *Scoliodon* genus. This conclusion was further supported by a subsequent analysis of the published *S. laticaudus* control region sequences from previous studies. Finally, based on these conclusions, we used machine learning to derive a new identification method for the cryptic species. This approach may be useful for the discovery of new species or cryptic species in other organisms.

## 1. Introduction

The genus *Scoliodon* belongs to Carcharhiniformes, Carcharhinidae, and is a group of small placental sharks that live in offshore waters [1,2]. There are two reported valid species in this genus: *Scoliodon laticaudus* and *Scoliodon macrorhynchos*. They normally live in shallow coastal areas with rocky and muddy substrates in the tropics and subtropics, where they primarily feed on small benthic fish, cephalopods, and crustaceans [1,2,3]. The classification of *Scoliodon* has undergone several changes. In 1838, Müller and Henle named a newly found species of *S. laticaudus*, which marked the establishment of the genus *Scoliodon* [4]. In 1852, *S. macrorhynchos* was proposed by Bleeker [5]. However, there was a subsequent argument that it should be the synonym of *S. laticaudus*. Later, several new species of the genus *Scoliodon* were described but were eventually invalidated due to the argumentative consideration of the synonyms of *Scoliodon* species [6]. Therefore, *Scoliodon* has long been considered a monotypic genus. Then, White et al. [2] used morphological and molecular methods to reclassify *Scoliodon* and proposed three valid species: *S. laticaudus*, which lives along the coasts of India, Pakistan, and Sri Lanka; *S. macrorhynchos*, which lives along the western Pacific coast; and *Scoliodon muelleri* (first described by Müller et al. [7]), which mainly lives in the Bay of Bengal. *Scoliodon muelleri* has not been widely recognized due to a lack of sufficient evidence. Recently, Kean Chong Lim et al. [8] reclassified *Scoliodon* with an integrated taxonomic approach, identifying *S. laticaudus* and *S. macrorhynchos* with the Strait of Malacca as a spatial divider. *S.* cf. *laticaudus* living in the waters of the Strait of Malacca is actually a synonym of *S. laticaudus*. Distinguishing the species in *Scoliodon* is difficult because of the small differences in their morphological characteristics [2].

Traditional taxonomy strongly relies on the morphological characteristics of species [9], but morphology-based taxonomy has intractable drawbacks, e.g., the distinction of morphological characteristics may not be significant among closely related species. Therefore, DNA barcoding has gradually become a reliable tool in taxonomy [10,11]. Most modern molecular-based animal taxonomy studies use partial genes in the mitochondrial genome as molecular markers, which have remarkably contributed to the discovery of cryptic species of fish. Lara et al. [12] identified at least four new cryptic species via the *COI* gene of Cuban freshwater fish and reported that two different species might be essentially identical. Winterbottom et al. [13] revealed dozens of possible new species of the genus *Trimma* via DNA barcoding. With the development of molecular technologies, DNA barcode-based species identification methods are gradually becoming popular.

The mitochondrial genome is highly conserved, simple in structure, rapidly evolving, and maternally inherited [14,15,16], which makes it a good molecular marker for studying biological phylogeny and population polymorphism. Its commonly used marker genes are *COI*, *NADH2*, *Cytb*, etc. [17,18,19]. *COI* is the earliest and most commonly used molecular marker for animal phylogenetic studies [20,21]. When the number of species studied increases, information on the sequences of single and multiple mitochondrial gene fragments may no longer be sufficient. The complete mitochondrial genome has become a better tool. In fish-related studies, mitochondrial genomes are often used in areas such as fish phylogeny, genetic diversity, and differentiation time estimation [22,23,24].

Machine learning has been widely used as an effective tool in various biological studies [25,26], especially for functional structure prediction based on gene sequences and sequence classification. It has been proven to be very effective [27]. Deep learning using neural network models has become increasingly popular in recent years and has provided new insights into biological studies [28,29].

In this study, we collected a sample (*Scoliodon* sp.) of the genus *Scoliodon* from Ranong, Thailand, and obtained its complete mitochondrial genome. We found that the genetic distance was significantly greater than the intra-specific p-distance of the genus *Scoliodon*, which is close to the inter-specific p-distance. Phylogenetic analysis and divergence time analysis revealed that *Scoliodon* sp. was well separated from the other two species. This finding reveals that *Scoliodon* sp. is a potential new species of *Scoliodon*. Based on these findings, we used machine learning methods to classify cryptic species and initially obtained valid results. In future studies on cryptic species, genetic distance analysis using the entire mitochondrial genome of an organism or further species classification using machine learning methods may constitute a superior route if reliable results cannot be obtained via morphological or single-gene difference analysis alone.

## 2. Results

### 2.1. Genome Organization and Nucleotide Composition

**Figure 1:**
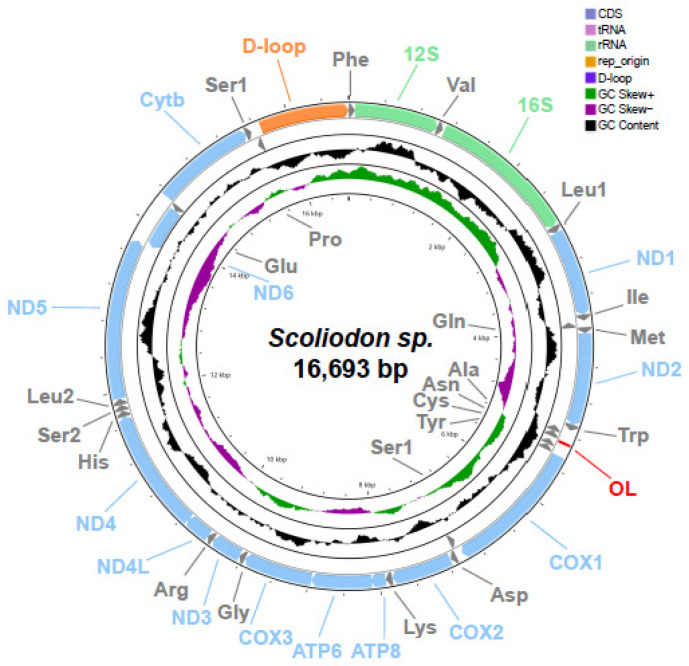
Graphical map of the mitochondrial genome of *Scoliodon* sp.

The complete mitochondrial genome of *Scoliodon* sp. is 16,693 bp in length. It includes 37 typical genes, 13 protein-coding genes (PCGs), 22 tRNA genes, 2 rRNA genes, and 1 non-coding region. The gene order and transcriptional direction are similar to those of a typical fish mitochondrial genome (Figure 1, Table S1). All genes are encoded on the heavy strand (H-strand) except for *NADH6* and eight tRNA genes (Gln, Ala, Asn, Cys, Thr, Ser, Glu, and Pro) on the light strand (L-strand). Most PCGs had a normal start codon (ATG), except for the *COI* gene, which uses GTG as the start codon, similar to other *Scoliodon* species. All PCGs are normal termination codons (TAA, TAG, or T-) except for ND1, which ends with AGA.

Compared with the 13 PCGs in the mitochondrial genome of *Scoliodon* species, those in the *Scoliodon* species presented high amino acid homology. The highest homology was found for the *ATP8* and *NADH4L* genes with 100% homology among these species. Moreover, the *COI* and *COII* genes of *Scoliodon* sp. were 100% similar to those of *S. laticaudus* and slightly less similar to those of *S. macrorhynchos*. The lowest homology in the *NADH5* gene was 97.21%, which was 97.21% similar between *Scoliodon* sp. and *S. macrorhynchos*.

### 2.2. Multi-Gene Association Genetic Distance Analysis of the Mitochondrial Genome

**Figure 2:**
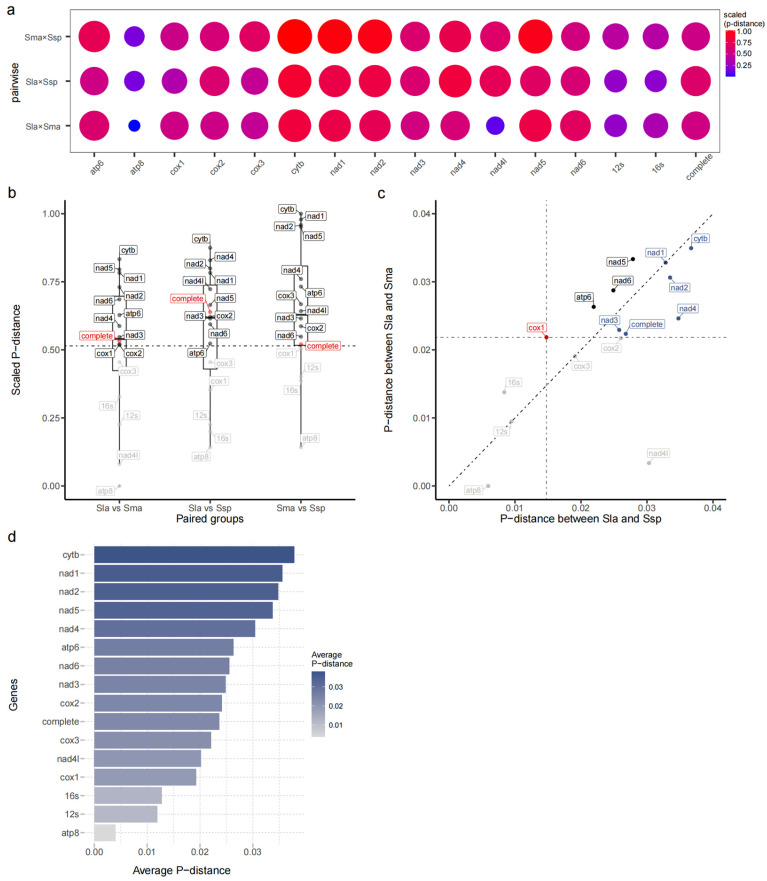
P-distance analyses of *Scoliodon* sp., *S. macrorhynchos*, and *S. laticaudus*, which are abbreviated as Ssp, Sma, and Sla, respectively. (**a**) Bubble chart of the p-distance of individual genes among Ssp, Sma, and Sla. The size and colour both represent the p-distance. (**b**) P-distance for each gene between every pair of the three species, the dashed line indicates the average P-distance; genes above this average are shown in black, while those below it are shown in gray. The data are scaled to highlight the differences. (**c**) Scatter plot of individual genes in terms of p-distance Sla × Ssp and Sla × Sma. The red spot represents the p-distance of the *COI*, which is commonly used for species identification. The grey dots indicate that the genes have shorter p-distances than *COI*. The blue and black spots represent genes with $Pdis_{Ssp\times Sla}$ values higher and lower than ${\mathrm{Pdis}}_{\mathrm{Sma}\times \mathrm{Sla}}$, respectively. (**d**) Average p-distance of different genes in the genus *Scoliodon*.

For ease of description, the p-distance analysis is denoted as *Pdis*, and the average p-distance is denoted as *Avg*. The p-distance analyses of *Scoliodon* sp., *S. macrorhynchos*, and *S. laticaudus* are abbreviated as Ssp, Sma, and Sla, respectively. In the complete mitochondrial genome of *Scoliodon*, $Pdis_{Ssp\times Sla}$ and $Pdis_{Ssp\times Sma}$ were slightly greater than $Pdis_{Sma\times Sla}$. For most of the 13 PCGs and 2 rRNA genes, $Pdis_{Ssp\times Sla}$ and $Pdis_{Ssp\times Sma}$ were also higher than $Pdis_{Sma\times Sla}$ or at least at the same level (Figure 2a). This result indicates that the average p-distance level between *Scoliodon* sp. and the other species is slightly greater than the inter-species p-distance (defined by $Pdis_{Sma\times Sla}$). Notably, for the *COI* genes, $Pdis_{Ssp\times Sla}$ was distinctly smaller than the inter-species p-distance (Figure 2b).

A scatter plot shows the genes that are at greater differences than the inter-specific differences between the two species (Figure 2b). Eight genes (*NADH1*, *NADH2*, *NADH3*, *NADH4*, *NADH5*, *NADH6*, *ATP6*, and *Cytb*) and the complete genome showed higher p-distance than *COI* in the comparison of p-distances for any pair of the three *Scoliodon* species (Figure S1). Among them, the $Pdis_{Ssp\times Sla}$ values of five genes and the complete genome are greater than or equal to $Pdis_{Sma\times Sla}$. The average p-distance across the three pairwise comparisons for each gene indicates that *Cytb*, *NADH1*, *NADH2*, *NADH5*, and *NADH* are the most divergent genes in *Scoliodon* (Figure 2c,d).

The mitochondrial genomes of the three *Scoliodon* species show no significant structural variation. Despite the lack of structural differences, the genetic distances suggest that there are significant differences between *Scoliodon* sp. and the two valid species, which supports the possibility that it is a new species.

### 2.3. Population Genetic Distance Analysis Based on the Control Region Sequence

**Figure 3:**
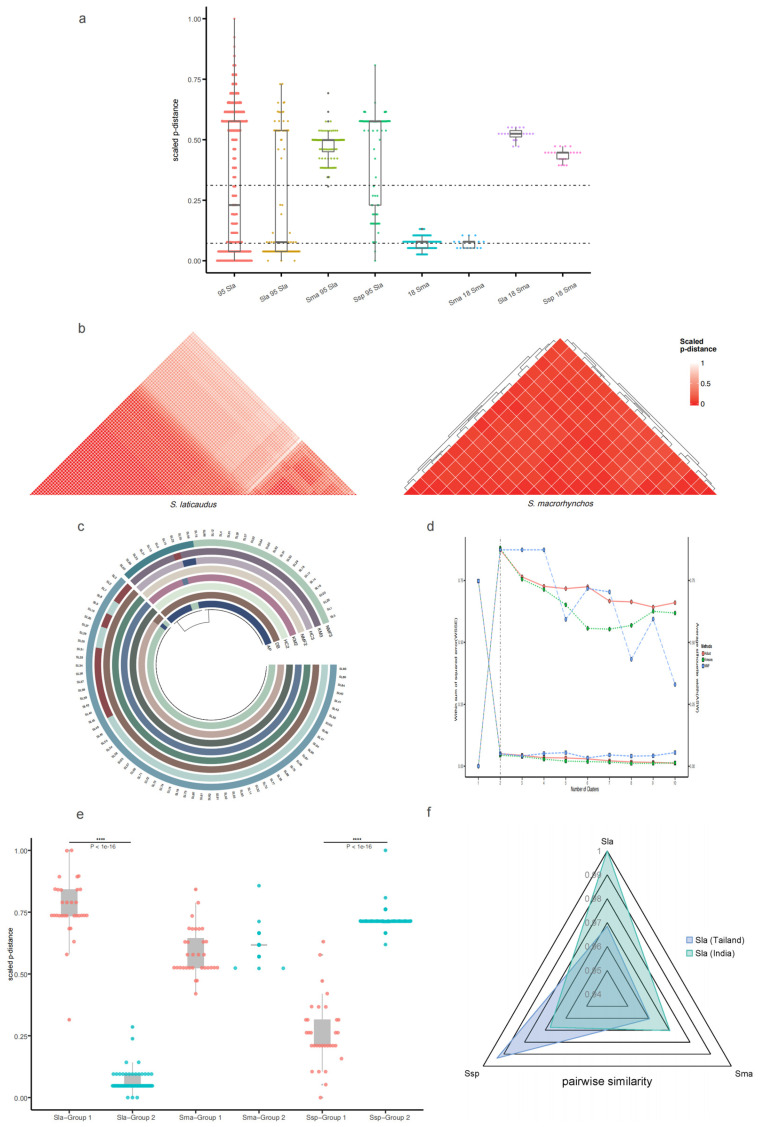
P-distance analysis of *S. macrorhynchos* (18 individuals) and *S. laticaudus* (95 individuals), which are abbreviated as Sma_18_ and Sla_95_, respectively. (**a**) Boxplot of the p-distances of Sla_95_, Sla_95_ × Sla, Sla_95_ × Sma, Sla_95_ × Ssp, Sma_18_, Sma_18_ × Sma, Sma_18_ × Sla, and Sma_18_ × Ssp. (**b**) Heatmap of the single-gene p-distance matrix for Sla and Sma; the colours indicate the p-distance (max: white; min: red). (**c**) Automatic clustering of Sma_18_ and Sla_95_ by five algorithms, identical colors represent the same cluster. (**d**) Determination of the optimal number of categories in cluster analysis using two evaluation metrics. (**e**) Boxplot of the p-distance of Sla_95_ (separated into two groups) with Sla, Sma, and Ssp. Five levels of significance were defined,**** for *p*-value < 1 × 10^−5^, P-values were caculated by the Wilcoxon rank-sum test. (**f**) Radar map of the pairwise similarity among different species.

$Pdis_{Sma18}$ remained slightly different from one another. This finding highlights the low intra-specific mutation rate of the control region within the genus *Scoliodon* (Figure 3). $Pdis_{Sma\times Sma18}$ generally showed a similar low p-distance to $Pdis_{Sma18}$. The same fragment of the *S. laticaudus* and *Scoliodon* sp. genomes differed more from the control region sequences of 18 *S. macrorhynchos*, and it shows a higher p-distance with similar $Avg\left(Pdis_{Ssp\times Sma18}\right)$ to $Avg\left(Pdis_{Sla\times Sma18}\right)$. The p-distance calculations for 95 control region sequences of *S. laticaudus* revealed a large variation (Figure 3a). Most of the p-distances showed similar lower levels at the intra-species level (defined by $Pdis_{Sma18}$) or similar higher levels at the inter-species level (defined by $Pdis_{Sla\times Sma18}$). $Pdis_{Sma\times Sla95}$ showed a greater degree of fluctuation relative to $Pdis_{\mathrm{Sma}\times Sma18}$ and generally remained at the inter-species level. $Pdis_{Ssp\times Sla95}$ and $Pdis_{Sla\times Sla95}$ showed similar larger differences than $Pdis_{Sla95}$. However, the data points for $Pdis_{Sla\times Sla95}$ were more concentrated on the low intra-species p-distance levels, and those for $Pdis_{Ssp\times Sla95}$ were more concentrated on the high inter-species p-distance levels.

The heatmap based on the p-distance matrix shows two separate low p-distance regions and a high p-distance region of 95 *S. laticaudus*. The results indicate that *S. laticaudus* can be divided into two groups with significant p-distance differences (Figure 3b). A part of 18 *S. macrorhynchos* served as a control with a more consistent low p-distance, which partially demonstrates the low intra-specific variability in the genus. In addition, the heatmap of the genetic distance matrix for the 86 *S. laticaudus*’s *COI* genes demonstrates consistent results. The genetic distance differences among *COI* genes are smaller and more consistent than those among the control region sequences, likely because protein-coding genes such as *COI* tend to be more conserved than non-coding regions, such as the control region.

### 2.4. Verification of the Grouping Results

In previous analyses, *S. laticaudus* was classified into different groups and defined as different species after the comparison of inter-species and intra-species p-distances. Five different algorithms were applied to clarify the clustering status of 95 *S. laticaudus*, and three different methods were applied to confirm the robustness of the algorithms’ results.

The three algorithms (NMF [30], K-means [31], and Hierarchical Clustering [32]) require the number of clusters to be specified. The results show that 95 control region sequences of *S. laticaudus* were divided into two clusters with almost identical clustering results or three clusters with more obvious differences. The other algorithms, which were DBSCAN (Density-Based Spatial Clustering of Applications with Noise) [33] and affinity propagation (AP) clustering [34], did not confirm the number of taxa before dividing into two clusters, and the classification is the same as that of the aforementioned algorithms (Figure 3c). The results show that the best number of clusters is two with an inflection point occurring under the sum of squared error (SSE) in the “Elbow” method [35] and average silhouette method [36] (Figure 3d). The gap statistic method does not reach the maximum gap at two clusters as real data, and the gap curve can have many local maxima [37]. Another large gap appeared when the data were clustered into 6 clusters, and the gap was at its maximum at 10 or more clusters. However, because of the very significant inflection point at two clusters, the number of well-separated clusters was considered to be two.

The control region fragments of the *Scoliodon* species were divided into two groups for the p-distance analysis, and a new boxplot is shown in Figure 3e. The p-distance of *S. laticaudus* and *S. macrorhynchos* in Group 1 was significantly greater than that at the inter-species level, whereas that of Group 2 was lower at the intra-species level. In contrast, Group 1 of *Scoliodon* sp. was at a lower p-distance level, and Group 2 was at a higher p-distance level.

The *NADH2* gene sequences of the *Scoliodon* species genome were compared with identical sequences of *S. laticaudus*, which was previously considered *S. muelleri* from India and Thailand. Pairwise similarity is defined as a value that is negatively correlated with the p-distance to demonstrate the sequence similarity between species. *Scoliodon laticaudus* (JQ518654) from India has a higher pairwise similarity to the *S. laticaudus* genome (NC_042504) and a low similarity to the genomes of *S. macrorhynchos* (NC_018052) and *Scoliodon* sp. (OL960037). Conversely, *S. laticaudus* (JQ518655) from Thailand has significantly greater similarity to the *Scoliodon* sp. genome and lower similarity to the other two species (Figure 3f).

### 2.5. Phylogenetic Analysis and Divergence Time Estimates of Genus Scoliodon

**Figure 4:**
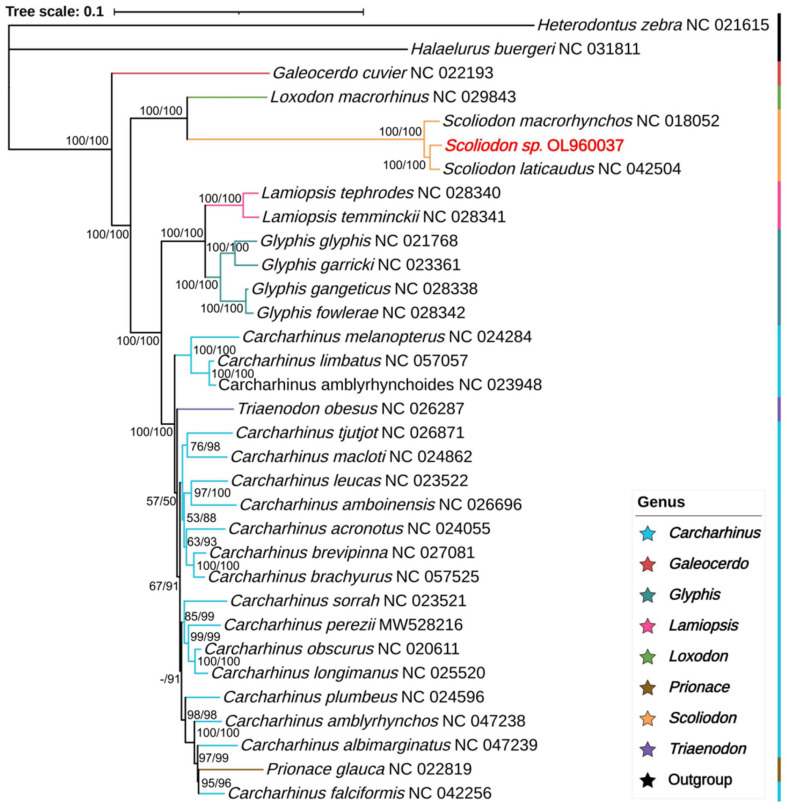
Phylogenetic tree of the family Carcharhinidae, which was inferred from twelve H-strand PCGs (excluding the third codon) and two rRNAs using the Bayesian inference (BI) and maximum likelihood (ML) methods. The numbers on the branches indicate the bootstrap (left) and posterior probabilities (right), and - indicates values lower than 50%. Branches in different colors represent different genera.

The phylogenetic analysis included eight genera of the family Carcharhinidae (*Carcharhinus*, *Galeocerdo*, *Glyphis*, *Lamiopsis*, *Loxodon*, *Prionace*, *Scoliodon*, and *Triaenodon*) (Figure 4). The posterior probability and bootstrap values that support most clades are high. In most clades, the ML values are greater than the BI values. The bootstrap support values of one clade are low (<50%), whereas the posterior probability is 91%.

The phylogenetic tree shows that the genera *Glyphis*, *Lamiopsis*, and *Scoliodon* were monophyletic. The genera *Prionace* and *Triaenodon* clustered with *Carcharhinus* species as sister groups, which is similar to previous results [38]. *Scoliodon* sp. clustered to *S. laticaudus* and formed a monophyletic group with *S. macrorhynchos*. The clade of the genus *Scoliodon* is longer than other clades. Then, the *Scoliodon* species clustered with the genera *Loxodon* and *Galeocerdo*, which are far from other Carcharhinidae. All species of Carcharhinidae clustered together with high support values (BI and ML), which suggests that these results are reliable.

Fossil-calibrated divergence time estimates indicate that the genus *Scoliodon* separated from the genus *Loxodon* approximately 77.33 mya (Figure S2). The oldest extant species in the genus is *S. macrorhynchos*, which appeared approximately 16.77 mya. Approximately 10.43 mya, *Scoliodon* sp. separated from *S. laticaudus* and formed *two distinct* branches. The divergence time between *Glyphis fowlerae* and *Glyphis gangeticus* in the genus *Glyphis* was approximately 5.18 mya, and the divergence time between *Carcharhinus limbatus* and *Carcharhinus amblyrhynchoides* in the genus *Carcharhinus* was approximately 6.75 mya. In terms of the divergence time alone, *Scoliodon* sp. diverged from *S. laticaudus* for a longer time than the two divergences.

### 2.6. Cryptic Species Classification Using Machine Learning

**Figure 5:**
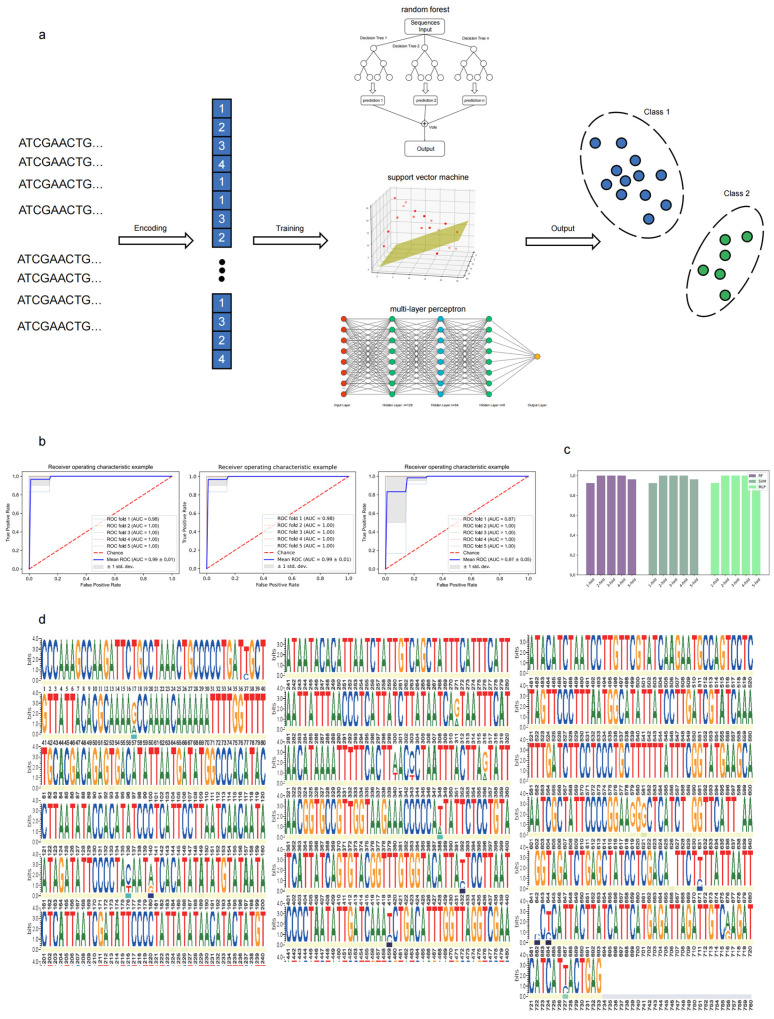
Identification of cryptic species using machine learning models. (**a**) Pipeline of machine learning for cryptic species identification. (**b**) ROC curves of the three models under the 5-fold cross-validation and AUC values. (**c**) AUC values for the three models. (**d**) Weblogo of the control region sequences of *S. laticaudus.* The importance of every site is shown in different colours under the weblogo.

The process based on the machine learning models for cryptic species identification is demonstrated in Figure 5a. The ROC curves of the three models under five-fold cross-validation were compared, and the AUC values were calculated (Figure 5b,c). AUC (area under the subject operating characteristic curve) is a measure of the model’s classification ability, and the closer the AUC value is to 1, the better the model’s ability to distinguish between different species. The results showed that the AUC values of the three models were all greater than 0.90, indicating that their classification predictions were more accurate on the dataset. The models can achieve accurate predictions on the datasets in this study. The random forest model and support vector machine model had consistent prediction accuracies and maintained stable accuracies in cross-validation. Although the multilayer perceptron model maintained an AUC mean greater than 0.9, the variance of AUC among different verification groups in the cross-validation was greater, so the model was relatively less robust on the datasets. Overfitting tends to occur because of the small amount of data, so it is difficult to achieve better robustness on the validation datasets. The F1 scores of the three models in the five-fold cross-validation were calculated and used to construct the confusion matrix (Figure S3).

The importance of the random forest model at different sequence sites was obtained. It theoretically reflects the contribution of different sites to the results of the model in the classification process, so it was used to find representative inter-species key sites of sequences and plot the full-length weblogo (Figure 5d). Most of the sequence sites are highly conserved and do not show inter-species differences. All sites with high importance are maintained with the sequence sites with large differences in the middle. However, not all differential sites are highly important. This finding proves that sequence differences may occur within the same species, and the model can effectively distinguish the inter-species and intra-species variations. Most sites had a significance of 0, a few (n = 39) sites had an importance value greater than 0, and only six sites had an importance value greater than 0.1. Notably, only a few sites in *S. laticaudus* and *Scoliodon* sp. sequences served as key inter-species variations, contributing most to species classification. This observed differentiation in key sites supports the hypothesis that *Scoliodon* sp. may represent a cryptic species within the genus, warranting further investigation.

## 3. Discussion

Using the conservation of mitochondria to classify species and determine their evolutionary status is a common approach in taxonomic studies. In particular, species identification based on *COI* gene sequences is widely used [17,39]. Some studies have shown that for closely related or understudied species, differences in *COI* genes may not be sufficient to reflect these differences [40]. In this study, the p-distance of the *COI* gene between *Scoliodon* sp. and *S. laticaudus* was lower than the inter-species level defined by the p-distance between *S. laticaudus* and *S. macrorhynchos*, so traditional molecular classification methods using *COI* genes cannot distinguish *Scoliodon* sp. from *S. laticaudus*. Mitochondrial genome-wide p-distance calculations showed that the distance between *Scoliodon* sp. and two valid *Scoliodon* species was greater than that between the two valid species. More than half of the genes had greater p-distances between *Scoliodon* sp. and *S. laticaudus*. This result implies that *Scoliodon* sp. may be a new species of the genus *Scoliodon*.

To further illustrate the relationship of the genus *Scoliodon* and verify that *Scoliodon* sp. is considered a cryptic species, we used multiple sequence data from the published mitochondrial control regions of *S. laticaudus* and *S. macrorhynchos* in previous studies. The p-distances between the sequences revealed significant heterogeneity among the control region sequences of *S. laticaudus* and the possible existence of cryptic species in different *S. laticaudus* populations. The subsequent cluster analysis proves that *S. laticaudus* can be classified into two classes at the optimal classification level. A comparison of the p-distances of the two classes of the genus *Scoliodon* revealed that Group 1 presented greater p-distances from *S. laticaudus* and *S. laticaudus* in a previous study, which most likely resulted from the mixing of *S. laticaudus* and *Scoliodon* sp. These analyses further confirm the possibility that *Scoliodon* sp. is a cryptic species similar to *S. laticaudus*.

Then, we conducted a phylogenetic and divergence time analysis that combined the published complete genomes of Carcharhinidae and several pieces of fossil evidence. The results show that *S. laticaudus* and *Scoliodon* sp. can be divided into two branches. The divergence time of these two species is sufficient for both to become independent species compared with other late-forming species of Carcharhinidae.

Although no significant structural variation was detected in the mitochondrial genomes of the three *Scoliodon* species, the genetic distance and phylogenetic analyses provided evidence of differences between *Scoliodon* sp. and the other two valid species, and these data consistently support the unique status of *Scoliodon* sp. This finding highlights the importance of integrating multiple analytical approaches to draw reliable conclusions about species boundaries.

In 2010, White et al. [2] resurrected *S. macrorhynchos* and divided *S. macrorhynchos* and *S. laticaudus* from India and *S. laticaudus* from Thailand into three separate clades via a phylogenetic analysis based on the *NADH2* gene. *S. laticaudus* from Thailand became the base sister species of the other two species. Based on Müller’s description of *S. muelleri* [7], White believes that *S. laticaudus* from the Bay of Bengal is actually *S. muelleri*. According to *An Atlas on the Elasmobranch Fishery Resources of India* [41], *S. laticaudus* has a large distribution on the west coast of India and very little distribution on the east coast; thus, the difference in catches between the east and west coasts is a staggering 73-fold. Combining geographic factors and the fact that *Scoliodon* generally lives in shallow waters, White suggested that the narrow shelf of the east coast of India or southern Sri Lanka contributed to the geographic isolation. Through multiple sequence comparisons, we found that *Scoliodon* sp. and *S. laticaudus* in this study had extremely high similarity to *S. laticaudus* from approximately the same area as White’s results. However, due to a lack of sufficient evidence, among other reasons, the species has not yet gained widespread acceptance, so there are still only two active species in the genus Scoliodon. The findings of this study provide only preliminary evidence in support of the *S. muelleri* hypothesis proposed by White, and further studies will help to validate the taxonomic status. The cross-comparison similarity of different areas was relatively low, so we consider that the *Scoliodon* sp. in this study is likely the same species as the *S. muelleri* proposed by White.

We separately trained several machine learning models (including random forest, support vector machine, and multilayer perceptron) based on the existing control region sequences and provided new convenient and reusable cryptic species classification methods that do not depend on specific gene fragments. Each model was trained to obtain a classification model with high accuracy (ROC > 95) using up to 80% of the available current samples. This study provides preliminary evidence of the applicability of machine learning methods to discover cryptic species based on sequence information. However, while all three models performed well, achieving complete accuracy across all predicted results was challenging. This phenomenon may be more obvious when the verification datasets are enlarged due to the current small amount of training data, short training sequence lengths, or less sequence information in the selected training gene fragments.

In addition, it identified key variant nucleotide sites in the control region that affected the classification. On one hand, the model may need a longer input sequence (such as the complete mitochondrial sequence) to include more key sequence information. This approach is beneficial to the classification of cryptic species. On the other hand, if the model is used in large-scale datasets, it can reduce the complexity of the model by using only effective fragments that contain more differential sites. This approach can improve the calculation efficiency of the model and reduce the difficulty of collecting experimental data. This approach helps expand the application of the model. However, our model may not be sufficiently robust because of the lack of sequence data for *S. laticaudus* and *Scoliodon* sp., especially for protein-coding genes. We will attempt to collect more data in future studies or use methods such as data augmentation to achieve a more robust model trained on small samples. In addition, only ordinal encoding was used in this study. 

Deep learning models with more neural network layers are not used in the model because of the limited data volume. In the future, we will test different encoding methods (such as k-mer and sequence embedding based on representation learning) to determine the best encoding method. To improve the classification accuracy or simplify the training difficulty, more deep learning models and unsupervised learning models should be explored for cryptic species identification. Machine learning has the potential for wider application in species identification. The application of current methods to different species and mitochondrial gene sequence fragments and the use of migration learning to obtain models with greater generalizability are feasible research directions.

It is important to recognize the inherent limitations of identifying a new species based on a single individual. Although data collected from that individual provide important insights, they do not fully represent the genetic, morphological, and ecological diversity that may exist in the species. The population sequences that we obtained from NCBI strengthen the credibility of our results, but the short length of individual genes and the lack of substantial morphological evidence remain problematic. Thus, although our results suggest the possibility of a new species, further studies with larger sample sizes are necessary to confirm these results and eliminate potential biases from analysing individual samples. We encourage future studies to build on this preliminary work by expanding the dataset and employing additional methods to confirm our conclusions.

This study provides new insights into the phylogenetic relationships of *Scoliodon* and challenges the status of having only two valid species in the genus. The taxonomic approach in this study provides new ideas for the molecular identification of cryptic species in this genus. This approach may be useful for the discovery of new species and the identification of cryptic species in other organisms.

## 4. Materials and Methods

### 4.1. Specimen Collection, DNA Extraction, PCR Amplification, and Sequencing

One specimen of *Scoliodon* sp. was collected from Ranong, Thailand, and preserved at South China Agriculture University (voucher FJXM20120509-11). Genomic DNA was extracted from the gills using the TIANamp Marine Animals DNA Kit—TianGen (DP324, Beijing, China). The segments were amplified using Takara Ex Taq™ Version 2.0 plus. The parameters of the PCRs were mostly in accordance with the manufacturer’s recommendations. The fragments were amplified using sharks’ universal primers, which were designed by the published Carcharhinidae mitogenomes of the National Center of Biotechnology Information (NCBI) (https://www.ncbi.nlm.nih.gov (accessed on 3 April 2023)). The PCR products were sequenced using Sanger Dideoxy Sequencing; this method has an extremely low error rate of 0.1%, i.e., an error of only 1 of 1000 bp.

### 4.2. Sequence Assembly, Annotation, and Basic Analysis

Sequence data were analysed and compiled to create complete mitogenomes using the SeqMan program via the DNAStar v7.1 program [42]. Species identification was performed based on the morphological characteristics and fragments of the *COI* gene downloaded from NCBI. The mitogenome was first annotated with the online MITOS2 Web Server [43]. Then, the secondary structures of the tRNA genes were identified using the tRNAscan-SE Search Server v2.0 [44] and confirmed using Arwen v1.2 [45]. The annotation and accuracy of the boundary determination of protein-coding and ribosomal RNA genes were assessed through comparisons with other released reference mitogenomes of Carcharhinidae species after manual alignment using DNAman v6.0 [46]. The mitogenome of *Scoliodon* sp. was drawn into a full circular genome using the CGView Server v1.0 [47]. The studied shark mitogenome was uploaded to GenBank using the BankIt program (Genbank accession number: OL960037). Subsequently, we investigated potential structural variations in the mitochondrial genome, including gene rearrangements, duplications, and deletions. However, no significant structural variation was found between the two valid species (*S. laticaudus* and *S. macrorhynchos*) and the new putative species (*Scoliodon* sp.).

### 4.3. Genetic Distance Analysis

To determine the classification relationship of *Scoliodon* sp., the complete mitochondrial genome sequences and many single-gene sequences of two valid species of *Scoliodon* were obtained from NCBI, and the specific sequence information is shown in Table 1. In total, 3 complete mitochondrial genomes of the genus *Scoliodon* (*Scoliodon* sp., *S. laticaudus*, and *S. macrorhynchos*), 13 protein-coding genes, and 2 rRNA genes in the mitochondrial genomes were used for genetic distance calculation and visualization. The control region sequences of 18 *S. macrorhynchos* and 95 *S. laticaudus* were also used for the genetic distance calculations to distinguish different levels of genetic distance variation within and among species. Afterwards, to confirm the best clustering of 95 control region sequences of *S. laticaudus*, 5 methods (NFM, K-means, HC, DB, and AP) were applied to compare different numbers of clusters. Finally, two *NADH2* sequences from White et al. were used to confirm the relationship between *Scoliodon* sp. and *S. muelleri*.

For ease of description, species names are sometimes presented in abbreviated form. *S. laticaudus* is abbreviated as Sla, *S. macrorhynchos* is abbreviated as Sma, *Scoliodon* sp. is abbreviated as Ssp, the population of 95 *S. laticaudus* individuals is abbreviated as Sla_95_, and the population of 18 *S. macrorhynchos* individuals is abbreviated as Sma_18_. For the same reason, the representation of p-distances also uses the abbreviated form. For example, $Pdis_{A\times B}$ is the p-distance between A and B, $Pdis_{A18}$ is the p-distance between each two of the 18 As (306 data points in total), $Pdis_{B\times A18}$ is the p-distance between B and each A (18 data points in total), and $Avg\left(Pdis_{B\times A18}\right)$ is the average p-distance of $Pdis_{B\times A18}$. Information on the reference sequences applied in this study is shown in Table 1.

The genetic distances are presented as p-distances. The number of base differences per site between sequences is shown. All ambiguous positions were removed for each sequence pair (pairwise deletion option). The P-distances were determined via MEGA X [48], visualized in R v4.1.3 [49] using ggplot2 v3.3.6 [50], and preprocessed by the R package tidyr v1.2.1 [51] before visualization. For clarity of figures, the p-distance was scaled into 0–1 using the following formula:

$$Sacle\left(D_{i}\right)=\frac{D_{i}-Min\left(D\right)}{Max\left(D\right)-Min\left(D\right)}$$

*D_i_* is the genetic distance in sample *i*.

The control region p-distance was visualized using ComplexHeatmap v2.10.0 [52], ggrepel v0.9.1 [53], ggbeeswarm v0.6.0 [54], and ggthemes v4.2.4 [55]. Then, the p-distances were clustered using the *nmf* command in the NMF v0.24.0 package [56], which specifies the NMF algorithm as “brunet” and sets the number of runs to perform as 1000; the *hclust* command in R v4.1.3, which sets the agglomeration method to be used as “complete”; the *kmeans* command in R v4.1.3, which uses the default parameters; the apcluster command in the package apcluster v1.4.10 [57], which uses the default parameters; and the dbscan command in the package dbscan v1.1.11 [58], which sets the size (radius) of the epsilon neighbourhood as 0.5 and the number of minimum points required in the eps neighbourhood for core points (including the point itself) as 20. Clusters from different methods were visualized using the R package circlize v0.4.15 [59], factoextra v1.0.7 [60], and ggpol v0.0.7 [61]. The algorithms were compared through the sum of squared errors via the following formula:

$$SSE=\overset{K}{\underset{i=1}{\sum }}\underset{P\in C_{i}}{\sum }{\left|p-m_{i}\right|}^{2}$$

*K* is the number of clusters, *Ci* is cluster *i* with current *k*, *p* is a specimen in cluster *Ci*, and *mi* is the centre of *Ci*.

The silhouette coefficient is calculated as follows [36]:

(1) $$a\left(i\right)=\frac{1}{\left|C_{i}-1\right|}\sum_{j\in C_{i},i\neq j}d\left(i.j\right)$$

(2) $$b\left(i\right)=\underset{k\neq 1}{\min}\frac{1}{C_{k}}\sum_{j\in C_{k}}d\left(i,j\right)$$

(3) $$s\left(i\right)=\left\{\begin{array}{cc} \frac{b\left(i\right)-a\left(i\right)}{\max\left(a\left(i\right),b\left(i\right)\right)}, & \;\;if\left|C_{i}\right|>1\\ 0, & \;\;if\left|C_{i}\right|>1\;\; \end{array}\right.$$

*K* is the number of clusters, *C_i_* is cluster *i* with current *k*, and *d* (*i*, *j*) is the distance between *i* and *j*.

The gap statistic [37] is calculated via the R package cluster v2.1.4 [62] command clusGap, where the number of Monte Carlo (“bootstrap”) samples was set to 1000.

### 4.4. Phylogenetic Analyses and Divergence Time Analyses

In total, 31 full mitogenomes of Carcharhinidae were downloaded from NCBI. *Heterodontus zebra* (NC021615) and *Halaelurus buergeri* (NC031811) were used as outgroups. A partition approach was applied, and we distinguished three partitions: the first and second codons of 12 H-strand-encoded PCGs (excluding the *ND6* gene) and the two rRNA genes. The rRNAs and 12 PCGs were aligned via the MAFFT v7 and MACSE v2.03 programs [63,64], respectively. The ambiguously aligned fragments of the rRNAs were subsequently removed using Gblocks 0.91b [65]. The final dataset was created by concatenating these three segments using Phylosuite v1.2.2 [66].

ModelFinder was used to select the best-fit partition model using the greedy algorithm [67], and the GTR+F+I+G4 model was selected as the optimal model according to the AICc criterion. Phylogenetic analyses were performed using BI and ML analyses [68,69]. Confidence in the ML was assessed with bootstrapping under 10,000 ultrafast bootstraps using an approximate Bayes test by IQ-TREE v1.6.2 [70]. Bayesian inference was conducted in MrBayes v3.2.6 [71] under 2 parallel runs and 1,000,000 generations, and the initial 25% of the sampled data was discarded as burn-in with default settings. The tree dataset files were visualized using iTOL v6 [72].

The divergence times were estimated using BEAST v2.7.1 based on the two gblocks and two rRNA genes of 31 Carcharhinidae species [73]. Using the optimized relaxed clock (ORC) [74] as the clock model and Yule model as the tree prior for taxon sets, five fossils (Table S2) were used to calibrate the time. The length of the MCMC chain was set to 108, whereas 10% of the samples were classified as burn-in by TreeAnnotator. Tracer v1.7.2 [75] was subsequently used to confirm the output, and the Figtree v1.4.3 software was used to visualize the tree.

### 4.5. Machine Learning

A previous analysis showed that *Scoliodon* sp. could not be distinguished from *S. laticaudus* by *the COI* gene but could easily be distinguished by comparison with several other mitochondrial genome sequence fragments (e.g., the control region sequence, *NADH1*, and *NADH2*). Therefore, an accurate and robust identification method that can stably discover cryptic species such as *Scoliodon* sp. should be developed.

#### 4.5.1. Sequence Dataset Preprocessing

In total, 95 control region sequences of *S. laticaudus* were used as the inputs, which were first separated by strictly combining the clustering results (n-SL = 65; n-SP = 30), and only samples in the groups near *Scoliodon* sp. were set as *Scoliodon* sp. markers in all methods. All sequences were multi-sequence aligned using MAFFT [63] and encoded as follows: “A” for 1, “T” for 2, “C” for 3, “G” for 4, and “gap” for 0. Finally, the input matrix size was 95 × 733.

#### 4.5.2. Model Development

Various machine learning techniques were used to develop three models, including random forest (RF) [76], support vector machine (SVM) [77,78] and multilayered perceptron (MLP) [79,80]. All models were built based on sklearn v1.1.2 [81] in Python v3.9.5.

RF is a supervised learning method that involves combining multiple decision trees to make predictions. Each tree is trained on a different sub-dataset, and the final prediction is obtained by taking the average of the outputs from all trees. This technique helps reduce the variance associated with individual decision trees and yields more accurate and stable predictions [76,82,83].

SVMs are popular methods for supervised learning and are often used for classification or regression tasks. This technique involves transforming the training data into a higher-dimensional space and finding a linear boundary that separates the classes with the greatest margin while minimizing the distance between the boundary and the points on either side [77,82,83].

An MLP is a type of supervised machine learning algorithm that is often used for classification tasks. It consists of three layers: an input layer that takes in the input data, a hidden layer that has these data and computes complex relationships among the inputs, and an output layer that generates the final result. The learning process is terminated when the error rate becomes sufficiently small, and we used stochastic gradient descent to optimize the log-loss function [79,82,84].

#### 4.5.3. Model Evaluation

To avoid overfitting, 5-fold cross-validation was applied to the data. This process involves randomly dividing the data into five subsets and using each subset in turn as the testing set, whereas the remaining subsets are used for training. The results from the five-fold cross-validation are the average values of the accuracy obtained from the five tests. Different metrics such as precision, recall, and the F1 score, were used to evaluate the performance of each model. These parameters are defined as follows:

(4) $$Precision=\frac{True\;positives}{True\;positives+False\;positives}$$

(5) $$Recall=\frac{True\;positives}{True\;positives+False\;positives}$$

(6) $$F1-score=\frac{2*Precision*Recall}{Precision+Recall}$$

In addition, the performance of each model was visualized in a confusion matrix, and an AUC curve was drawn using Matplotlib v3.5.1 [85].

#### 4.5.4. Feature Importance

To evaluate how each model works, an RF with optimized hyperparameters was used to analyse the feature importance. The result was calculated with all 95 sequences as the input, and the feature importance was associated with each site in the CR sequences. The result is shown in the weblogo with the single-line heatmap. The weblogo was built by Python package WebLogo v3.7.12 [86], and the heatmap was drawn based on the Python package seaborn v0.12.1 [87].

## Figures and Tables

**Table 1:** Information on the reference sequences in the analyses.

Sequence Name	GenBank ID	Region
Complete mitochondrial genome of S. laticaudus	NC_042504	Complete mitochondrial genome
Complete mitochondrial genome of *S. macrorhynchos*	NC_018052	Complete mitochondrial genome
95 control region sequences of *S. laticaudus*	MW974731-MW974825	Control regions
115 *COI* sequences of *S. laticaudus*	MW974616-MW974730	*COI* genes
18 control region sequences of *S. macrorhynchos*	KX657688-KX657705	Control regions
*ND2* sequence of *S. laticaudus* (India ^1^)	JQ518654	*ND2* gene
*ND2* sequence of *S. laticaudus* (Thailand ^2^)	JQ518655	*ND2* gene

To distinguish identical genes obtained from samples of the same species in different locations, the species name is followed by the sampling location in the article.

## Data Availability

The genome reported in this paper has been deposited in the GenBank database under accession number OL960037.

## Supplementary Materials

The following supporting information can be downloaded at https://www.mdpi.com/article/10.3390/ijms252111851/s1.

## References

[1] Compagno L.J.V. (1984). Sharks of the world: An annotated and illustrated catalogue of shark species known to date. Part 2. Carcharhiniformes.

[2] White W., Last P., Naylor G. (2010). Scoliodon macrorhynchos (Bleeker, 1852), a second species of spadenose shark from the Western Pacific (Carcharhiniformes: Carcharhinidae). Descr. New Sharks Rays Borneo CSIRO Mar. Atmos. Res. Pap..

[3] Setna S., Sarangdhar P. (1948). Description, bionomics and development of Scoliodon sorrakowah (Cuvier). Rec. Zool. Surv. India.

[4] Müller J. (1837). Gattungen der Haifische und Rochen nach einer von ihm mit Hrn. Henle unternommenen gemeinschaftlichen Arbeit über die Naturgeschichte der Knorpelfische. Berichte Der Königlichen Preuss. Akad. Der Wiss. Zu Berl..

[5] Bleeker P. (1852). Bijdrage tot de Kennis der Plagiostomen van den Indischen Archipel.

[6] Springer V.G. (1964). A revision of the carcharhinid shark genera Scoliodon, Loxodon, and Rhizoprionodon. Proc. United States Natl. Mus..

[7] Müller J., Henle J. (1841). Systematische beschreibung der Plagiostomen.

[8] Lim K.C., White W.T., Then A.Y., Naylor G.J., Arunrugstichai S., Loh K.-H. (2022). Integrated taxonomy revealed genetic differences in morphologically similar and non-sympatric Scoliodon macrorhynchos and S. laticaudus. Animals.

[9] Austin J.J., Melville J. (2006). Incorporating historical museum specimens into molecular systematic and conservation genetics research. Mol. Ecol. Notes.

[10] Hebert P.D., Gregory T.R. (2005). The promise of DNA barcoding for taxonomy. Syst. Biol..

[11] Rach J., DeSalle R., Sarkar I.N., Schierwater B., Hadrys H. (2008). Character-based DNA barcoding allows discrimination of genera, species and populations in Odonata. Proc. R. Soc. B Biol. Sci..

[12] Lara A., Ponce de León J.L., Rodriguez R., Casane D., Cote G., Bernatchez L., García-Machado E. (2010). DNA barcoding of Cuban freshwater fishes: Evidence for cryptic species and taxonomic conflicts. Mol. Ecol. Resour..

[13] Winterbottom R., Hanner R.H., Burridge M., Zur M. (2014). A cornucopia of cryptic species-a DNA barcode analysis of the gobiid fish genus Trimma (Percomorpha, Gobiiformes). ZooKeys.

[14] Wolstenholme D.R. (1992). Animal mitochondrial DNA: Structure and evolution. Int. Rev. Cytol..

[15] Boore J.L. (1999). Animal mitochondrial genomes. Nucleic Acids Res..

[16] Curole J.P., Kocher T.D. (1999). Mitogenomics: Digging deeper with complete mitochondrial genomes. Trends Ecol. Evol..

[17] Kartavtsev Y.P. (2011). Divergence at Cyt-b and Co-1 mtDNA genes on different taxonomic levels and genetics of speciation in animals. Mitochondrial DNA.

[18] Zhu Z.Y., Yue G.H. (2008). The complete mitochondrial genome of red grouper Plectropomus leopardus and its applications in identification of grouper species. Aquaculture.

[19] Ma C., Cheng Q., Zhang Q., Zhuang P., Zhao Y. (2010). Genetic variation of Coilia ectenes (Clupeiformes: Engraulidae) revealed by the complete cytochrome b sequences of mitochondrial DNA. J. Exp. Mar. Biol. Ecol..

[20] Hebert P.D., Cywinska A., Ball S.L., DeWaard J.R. (2003). Biological identifications through DNA barcodes. Proc. R. Soc. London. Ser. B Biol. Sci..

[21] Souza H., Marchesin S., Itoyama M. (2016). Analysis of the mitochondrial COI gene and its informative potential for evolutionary inferences in the families Coreidae and Pentatomidae (Heteroptera). Genet. Mol. Res..

[22] Von der Heyden S., Lipinski M.R., Matthee C.A. (2010). Remarkably low mtDNA control region diversity in an abundant demersal fish. Mol. Phylogenetics Evol..

[23] Wang C., Chen H., Tian S., Yang C., Chen X. (2020). Novel gene rearrangement and the complete mitochondrial genome of Cynoglossus monopus: Insights into the envolution of the family Cynoglossidae (Pleuronectiformes). Int. J. Mol. Sci..

[24] Chen X., Wang J.-J., Ai W.-M., Chen H., Lin H.-D. (2018). Phylogeography and genetic population structure of the spadenose shark (Scoliodon macrorhynchos) from the Chinese coast. Mitochondrial Dna Part A.

[25] Boyko N., Kmetyk-Podubinska K., Andrusiak I. (2021). Application of Ensemble Methods of Strengthening in Search of Legal Information. Proceedings of the 2021 International Scientific Conference “Intellectual Systems of Decision Making and Problem of Computational Intelligence”.

[26] Reel P.S., Reel S., Pearson E., Trucco E., Jefferson E. (2021). Using machine learning approaches for multi-omics data analysis: A review. Biotechnol. Adv..

[27] Libbrecht M.W., Noble W.S. (2015). Machine learning applications in genetics and genomics. Nat. Rev. Genet..

[28] LeCun Y., Bengio Y., Hinton G. (2015). Deep learning. Nature.

[29] Angermueller C., Pärnamaa T., Parts L., Stegle O. (2016). Deep learning for computational biology. Mol. Syst. Biol..

[30] Lee D.D., Seung H.S. (1999). Learning the parts of objects by non-negative matrix factorization. Nature.

[31] MacQueen J. (1967). Classification and analysis of multivariate observations. In Proceedings of 5th Berkeley Symposium on Mathematical Statistics and Probability.

[32] Aggarwal C., Reddy C. (2013). Data Clustering Algorithms and Applications.

[33] Ester M., Kriegel H.-P., Sander J., Xu X. A density-based algorithm for discovering clusters in large spatial databases with noise. Proceedings of the 2nd International Conference on Knowledge Discovery and Data Mining.

[34] Frey B.J., Dueck D. (2007). Clustering by passing messages between data points. Science.

[35] Bholowalia P., Kumar A. (2014). EBK-means: A clustering technique based on elbow method and k-means in WSN. Int. J. Comput. Appl..

[36] Hruschka E.R., de Castro L.N., Campello R.J. (2004). Evolutionary Algorithms for Clustering Gene-Expression Data. Proceedings of the 4th IEEE International Conference on Data Mining (ICDM’04).

[37] Tibshirani R., Walther G., Hastie T. (2001). Estimating the number of clusters in a data set via the gap statistic. J. R. Stat. Soc. Ser. B (Stat. Methodol.).

[38] Wang C., Lai T., Ye P., Yan Y., Feutry P., He B., Huang Z., Zhu T., Wang J., Chen X. (2022). Novel duplication remnant in the first complete mitogenome of Hemitriakis japanica and the unique phylogenetic position of family Triakidae. Gene.

[39] Huang Y., Bian C., Liu Z., Wang L., Xue C., Huang H., Yi Y., You X., Song W., Mao X. (2020). The first genome survey of the Antarctic Krill (*Euphausia superba*) provides a valuable genetic resource for polar biomedical research. Mar. Drugs.

[40] Krück N.C., Tibbetts I.R., Ward R.D., Johnson J.W., Loh W.K., Ovenden J.R. (2013). Multi-gene barcoding to discriminate sibling species within a morphologically difficult fish genus (Sillago). Fish. Res..

[41] Raje S., Sivakami S., Mohanraj G., Manojkumar P., Raju A., Joshi K. (2007). Atlas on the Elasmobranch fishery resources of India. CMFRI Spec. Publ..

[42] Burland T.G. (2000). DNASTAR’s Lasergene sequence analysis software. Bioinformatics Methods and Protocols.

[43] Bernt M., Donath A., Jühling F., Externbrink F., Florentz C., Fritzsch G., Pütz J., Middendorf M., Stadler P.F. (2013). MITOS: Improved de novo metazoan mitochondrial genome annotation. Mol. Phylogenetics Evol..

[44] Lowe T.M., Chan P.P. (2016). tRNAscan-SE On-line: Integrating search and context for analysis of transfer RNA genes. Nucleic Acids Res..

[45] Laslett D., Canbäck B. (2008). ARWEN: A program to detect tRNA genes in metazoan mitochondrial nucleotide sequences. Bioinformatics.

[46] Wang W. (2015). The Molecular Detection of Corynespora Cassiicola on Cucumber by PCR Assay Using DNAman Software and NCBI. Proceedings of the International Conference on Computer and Computing Technologies in Agriculture.

[47] Grant J.R., Stothard P. (2008). The CGView Server: A comparative genomics tool for circular genomes. Nucleic Acids Res..

[48] Kumar S., Stecher G., Li M., Knyaz C., Tamura K. (2018). MEGA X: Molecular evolutionary genetics analysis across computing platforms. Mol. Biol. Evol..

[49] Team R.C. (2013). R: A Language and Environment for Statistical Computing.

[50] Villanueva R.A.M., Chen Z.J. (2019). ggplot2: Elegant Graphics for Data Analysis.

[51] Wickham H., Henry L. (2020). Tidyr: Tidy messy data. R Package Version.

[52] Gu Z., Eils R., Schlesner M. (2016). Complex heatmaps reveal patterns and correlations in multidimensional genomic data. Bioinformatics.

[53] Slowikowski K. (2018). ggrepel: Automatically position non-overlapping text labels with “ggplot2.”. R package version 0.8.0. https://CRAN.R-project.org/package=ggrepel.

[54] Clarke E., Sherrill-Mix S. ggbeeswarm: Categorical scatter (violin point) plots. R package version 0.6.0. Retrieved from 2017. https://CRAN.R-project.org/package=ggbeeswarm.

[55] Arnold J.B., Daroczi G., Werth B., Weitzner B., Kunst J., Auguie B. (2019). ggthemes: Extra Themes, Scales and Geoms for ’ggplot2’; R package version 4.2.0. https://CRAN.R-project.org/package=ggthemes.

[56] Gaujoux R., Seoighe C. (2010). A flexible R package for nonnegative matrix factorization. BMC Bioinform..

[57] Bodenhofer U., Kothmeier A., Hochreiter S. (2011). APCluster: An R package for affinity propagation clustering. Bioinformatics.

[58] Hahsler M., Piekenbrock M., Doran D. (2019). dbscan: Fast density-based clustering with R. J. Stat. Softw..

[59] Gu Z., Gu L., Eils R., Schlesner M., Brors B. (2014). Circlize implements and enhances circular visualization in R. Bioinformatics.

[60] Kassambara A., Mundt F. (2017). Factoextra: Extract and visualize the results of multivariate data analyses. R Package Version.

[61] Tiedemann F. (2020). Ggpol: Visualizing Social Science Data with ’ggplot2’; R package version 0.0.7. https://CRAN.R-project.org/package=ggpol.

[62] Maechler M., Rousseeuw P., Struyf A., Hubert M., Hornik K. (2012). Cluster: Cluster analysis basics and extensions. R Package Version.

[63] Katoh K., Standley D.M. (2013). MAFFT multiple sequence alignment software version 7: Improvements in performance and usability. Mol. Biol. Evol..

[64] Ranwez V., Douzery E.J., Cambon C., Chantret N., Delsuc F. (2018). MACSE v2: Toolkit for the alignment of coding sequences accounting for frameshifts and stop codons. Mol. Biol. Evol..

[65] Talavera G., Castresana J. (2007). Improvement of phylogenies after removing divergent and ambiguously aligned blocks from protein sequence alignments. Syst. Biol..

[66] Zhang D., Gao F., Jakovlić I., Zou H., Zhang J., Li W.X., Wang G.T. (2020). PhyloSuite: An integrated and scalable desktop platform for streamlined molecular sequence data management and evolutionary phylogenetics studies. Mol. Ecol. Resour..

[67] Kalyaanamoorthy S., Minh B.Q., Wong T.K., Von Haeseler A., Jermiin L.S. (2017). ModelFinder: Fast model selection for accurate phylogenetic estimates. Nat. Methods.

[68] Nylander J.A., Ronquist F., Huelsenbeck J.P., Nieves-Aldrey J. (2004). Bayesian phylogenetic analysis of combined data. Syst. Biol..

[69] Sitnikova T. (1996). Bootstrap method of interior-branch test for phylogenetic trees. Mol. Biol. Evol..

[70] Minh B.Q., Schmidt H.A., Chernomor O., Schrempf D., Woodhams M.D., Von Haeseler A., Lanfear R. (2020). IQ-TREE 2: New models and efficient methods for phylogenetic inference in the genomic era. Mol. Biol. Evol..

[71] Ronquist F., Teslenko M., Van Der Mark P., Ayres D.L., Darling A., Höhna S., Larget B., Liu L., Suchard M.A., Huelsenbeck J.P. (2012). MrBayes 3.2: Efficient Bayesian phylogenetic inference and model choice across a large model space. Syst. Biol..

[72] Letunic I., Bork P. (2016). Interactive tree of life (iTOL) v3: An online tool for the display and annotation of phylogenetic and other trees. Nucleic Acids Res..

[73] Bouckaert R., Vaughan T.G., Barido-Sottani J., Duchêne S., Fourment M., Gavryushkina A., Heled J., Jones G., Kühnert D., De Maio N. (2019). BEAST 2.5: An advanced software platform for Bayesian evolutionary analysis. PLoS Comput. Biol..

[74] Douglas J., Zhang R., Bouckaert R. (2021). Adaptive dating and fast proposals: Revisiting the phylogenetic relaxed clock model. PLoS Comput. Biol..

[75] Rambaut A., Drummond A.J., Xie D., Baele G., Suchard M.A. (2018). Posterior summarization in Bayesian phylogenetics using Tracer 1.7. Syst. Biol..

[76] Breiman L. (2001). Random forests. Mach. Learn..

[77] Cortes C., Vapnik V. (1995). Support-vector networks. Mach. Learn..

[78] Suykens J.A. (2001). Support vector machines: A nonlinear modelling and control perspective. Eur. J. Control..

[79] Glorot X., Bengio Y. (2010). Understanding the Difficulty of Training Deep Feedforward Neural Networks. Proceedings of the Thirteenth International Conference on Artificial Intelligence and Statistics.

[80] Poole D.I., Goebel R.G., Mackworth A.K. (1998). Computational Intelligence.

[81] Pedregosa F., Varoquaux G., Gramfort A., Michel V., Thirion B., Grisel O., Blondel M., Prettenhofer P., Weiss R., Dubourg V. (2011). Scikit-learn: Machine learning in Python. J. Mach. Learn. Res..

[82] Lee Y.W., Choi J.W., Shin E.-H. (2021). Machine learning model for predicting malaria using clinical information. Comput. Biol. Med..

[83] Balasubramaniam S., Kumar K.S. (2022). Optimal Ensemble Learning Model for COVID-19 detection using chest X-ray images. Biomed. Signal Process. Control..

[84] Ahmed A.M., Aly S.F. (2019). Egyptian License Plates Recognition System Using Morphologial Operations and Multi Layered Perceptron. Proceedings of the International Conference on ICT in Our Lives, Alexandria, Egypt.

[85] Hunter J.D. (2007). Matplotlib: A 2D graphics environment. Comput. Sci. Eng..

[86] Crooks G.E., Hon G., Chandonia J.-M., Brenner S.E. (2004). WebLogo: A sequence logo generator. Genome Res..

[87] Waskom M.L. (2021). Seaborn: Statistical data visualization. J. Open Source Softw..

