# Supplementary material for: Potential Cryptic Diversity in the Genus Scoliodon (Carcharhiniformes: Carcharhinidae): Insights from Mitochondrial Genome Sequencing
Source: Int J Mol Sci. 2024 Nov 4;25(21):11851. doi: 10.3390/ijms252111851 (PMC11546983; doi:10.3390/ijms252111851)
Supplement: Supplementary file 1 [file ijms-25-11851-s001.zip › Table S1-S2.pdf]

Table S1. Features of the complete mitochondrial genome for *Scoliodon* sp..

**Region**	**Strand**	**Position**	**Size (bp)**	**Codon**		**Anti-codon**	**Intergenic spacer (bp)**	**Amino acid homology (%)**	
				**Star**	**Stop**			***S. laticaudus***	***S. macrorhynchos***
tRNA-*Phe*	H	1-70	70			GAA	0		
12S rRNA	H	72-1,024	953				0		
tRNA-*Val*	H	1,022-1,093	72			TAC	-3		
16S rRNA	H	1,113-2,764	1,652				0		
tRNA-*Leu*1	H	2,764-2,838	75			TAA	-1		
ND1	H	2,839-3,813	975	ATG	TAA		0	97.53	97.53
tRNA-*Ile*	H	3,814-3,883	70			GAT	0		
tRNA-*Gln*	L	3885-3,956	72			TTG	0		
tRNA-*Met*	H	3,957-4,025	69			CAT	0		
ND2	H	4,026-5,072	1,047	ATG	T-		0	97.99	97.41
tRNA-*Trp*	H	5,071-5,141	71			TCA	-2		
tRNA-*Ala*	L	5,143-5,211	69			TGC	0		
tRNA-*Asn*	L	5,212-5,284	73			GTT	0		
O_L	-	5, 289-5,318	30						
tRNA-*Cys*	L	5,319-5,385	67			GCA	0		
tRNA-*Tyr*	L	5,387-5,455	69			GTA	0		
COI	H	5,457-7,013	1,557	GTG	TAA		0	100.00	99.23
tRNA-*Ser*1 (	L	7,014-7,084	71			TGA	0		
tRNA-*Asp*	H	7,088-7,156	69			GTC	0		
COII	H	7,164-7,854	691	ATG	T-		0	100.00	99.57
tRNA-*Lys*	H	7,855-7,928	74			TTT	0		
ATP8	H	7,930-8,097	168	ATG	TAA		0	100.00	100.00
ATP6	H	8,088-8,771	684	ATG	TAA		-10	99.12	98.24
COIII	H	8,771-9,556	786	ATG	TAA		-1	99.62	99.62
tRNA-*Gly*	H	9,559-9,628	70			TCC	0		
ND3	H	9,629-9,979	351	ATG	T-		0	99.14	99.14
tRNA-*Arg*	H	9,978-10,047	70			TCG	-2		
ND4L	H	10,048-10,344	297	ATG	TAA		0	100.00	100.00
ND4	H	10,338-11,718	1,381	ATG	T-		-7	97.61	97.61
tRNA-*His*	H	11,719-11,787	69			GTG	0		
tRNA-*Ser*2	H	11,788-11,854	67			GCT	0		
tRNA-*Leu*2	H	11,855-11,926	72			TAG	0		
ND5	H	11,928-13,757	1,830	ATG	TAA		0	98.85	97.21
ND6	L	13,753-14,274	522	ATG	AGA		-5	98.27	98.84
tRNA-*Glu*	L	14,275-14344	70			TTC	0		
Cytb	H	14,347-15,492	1,146	ATG	TAG		0	99.48	99.74
tRNA-*Thr*	H	15492-15,561	70			TGT	-1		
tRNA-*Pro*	L	15,564-15,632	69			TGG	0		
Control region	H	15,632-16,693					0		

Table S2. Fossil times used in divergent time analysis

Species	Fossil time (Mya)
Carcharhinus falciformis	23.03 - 20.44
Carcharhinus brachyurus	23.03 - 13.82
Galeocerdo cuvier	23.0 - 20.4
Triaenodon obesus	23.0 - 13.8
Sphyrna zygaena	37.2 - 33.9
