# Supplementary figures and images for: Potential Cryptic Diversity in the Genus Scoliodon (Carcharhiniformes: Carcharhinidae): Insights from Mitochondrial Genome Sequencing
Source: Int J Mol Sci. 2024 Nov 4;25(21):11851. doi: 10.3390/ijms252111851 (PMC11546983; doi:10.3390/ijms252111851)

a

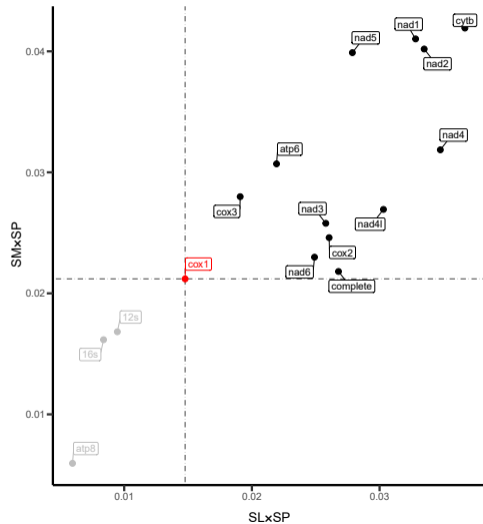

b

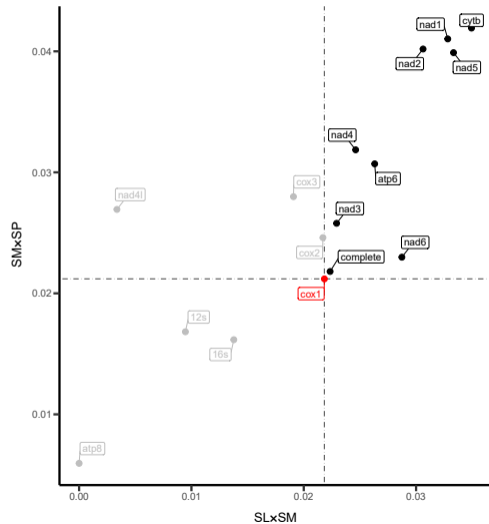

Supplement: Supplementary file 1 [file ijms-25-11851-s001.zip › fig s1 Comparative p-Distance Analysis of Three Scoliodon Species.pdf]

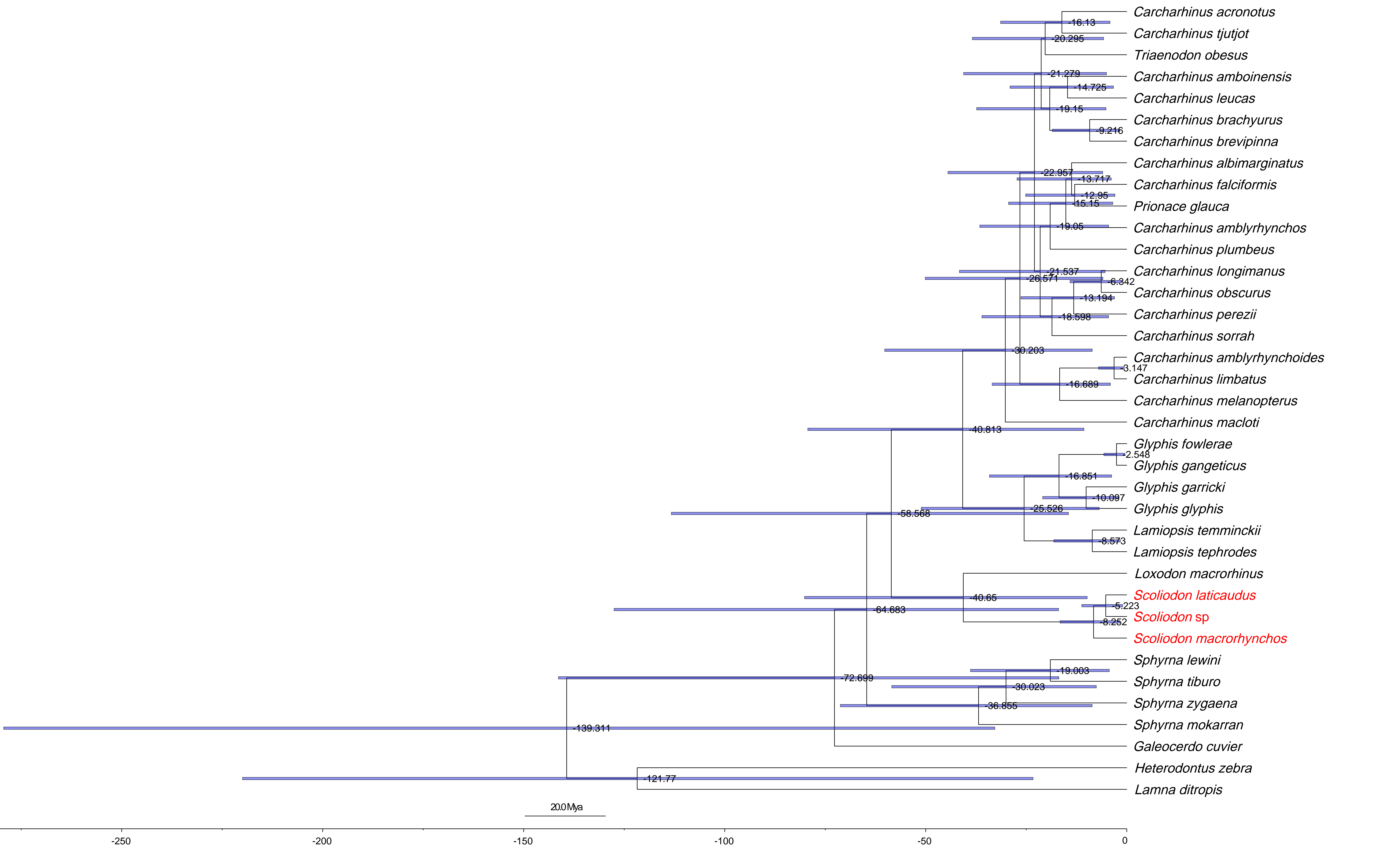

Supplement: Supplementary file 1 [file ijms-25-11851-s001.zip › fig s2 Fossil-Calibrated Divergence Time Estimates.pdf]

a

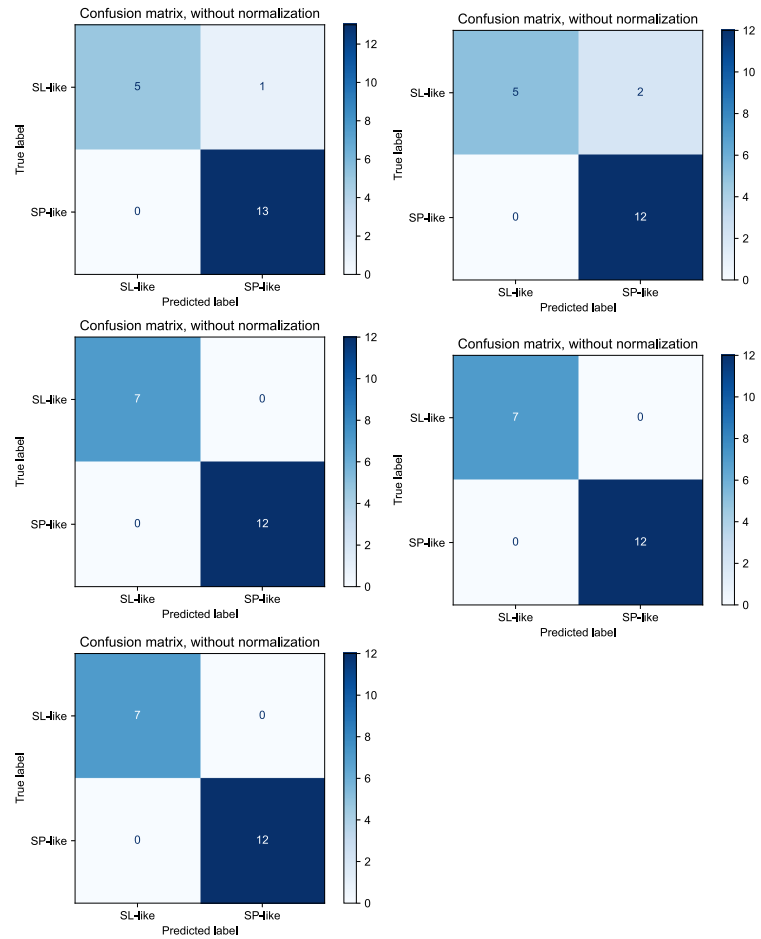

b

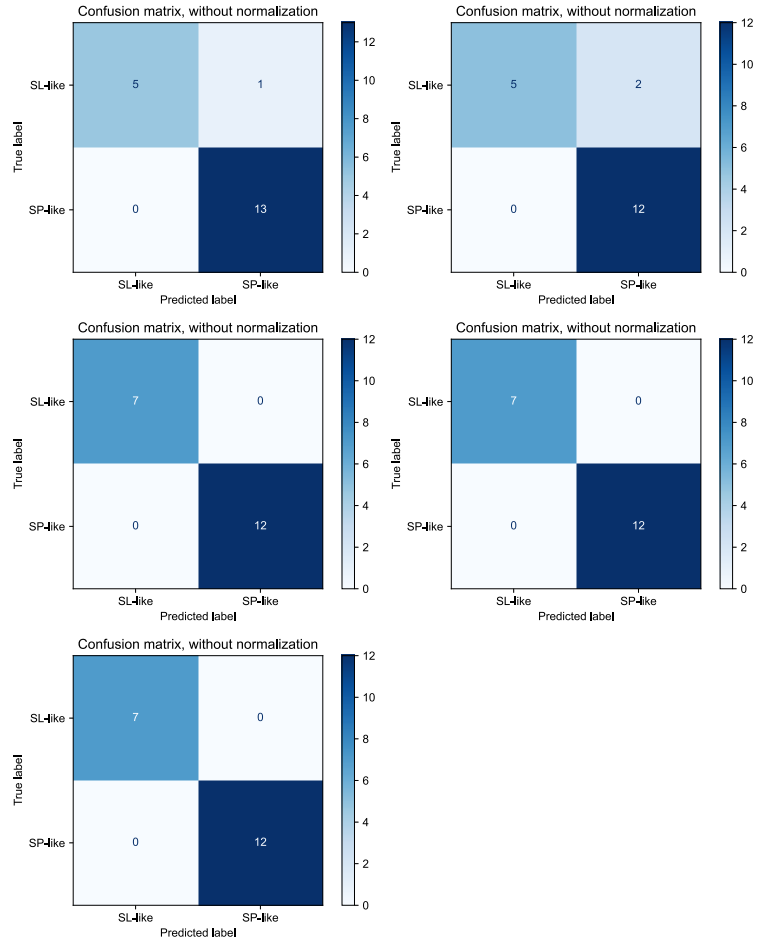

c

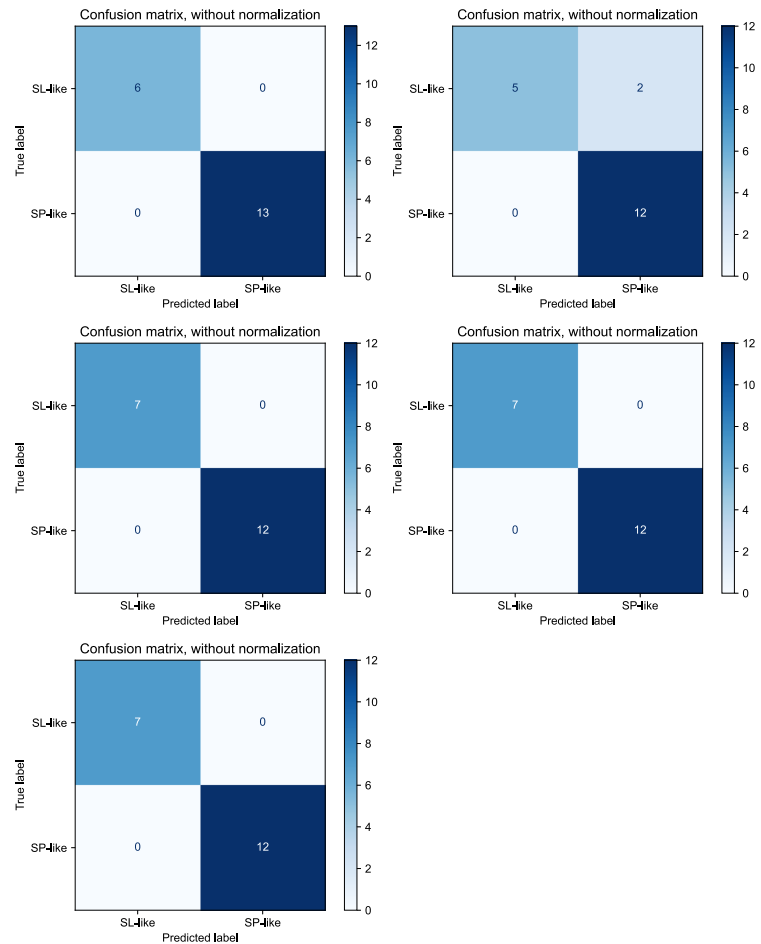

d

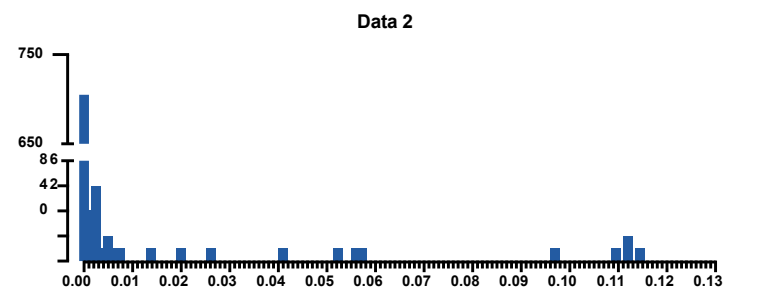

e

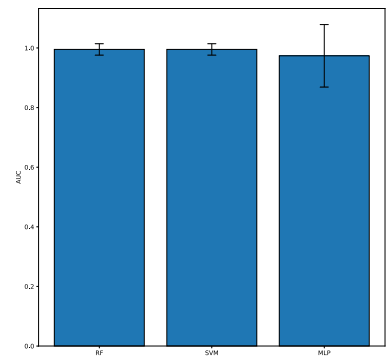

Supplement: Supplementary file 1 [file ijms-25-11851-s001.zip › fig s3 Confusion Matrix of the Three Models Based on F1 Scores from 5-Fold Cross-Validation.pdf]
